# Parallel re-modeling of EF-1α function: divergent EF-1α genes co-occur with EFL genes in diverse distantly related eukaryotes

**DOI:** 10.1186/1471-2148-13-131

**Published:** 2013-06-26

**Authors:** Ryoma Kamikawa, Matthew W Brown, Yuki Nishimura, Yoshihiko Sako, Aaron A Heiss, Naoji Yubuki, Ryan Gawryluk, Alastair GB Simpson, Andrew J Roger, Tetsuo Hashimoto, Yuji Inagaki

**Affiliations:** 1 Graduate School of Global Environmental Studies, Kyoto University, Kyoto 606-8501, Japan; 2 Graduate School of Human and Environmental Studies, Kyoto University, Kyoto 606-8501, Japan; 3 Centre for Comparative Genomics and Evolutionary Bioinformatics, Department of Biochemistry and Molecular Biology, Dalhousie University, Halifax, NS, Canada; 4 Graduate School of Life and Environmental Sciences, University of Tsukuba, 1-1-1 Tennoudai, Tsukuba, Ibaraki 305-8572, Japan; 5 Graduate School of Agriculture, Kyoto University, Kitashirakawa Oiwake-cho, Kyoto 606-8502, Japan; 6 Department of Biology, Dalhousie University, Halifax NS, Canada; 7 Department of Botany, University of British Columbia, 6270 University Blvd., Vancouver, BC V6T 1Z4, Canada; 8 Center for Computational Sciences, University of Tsukuba, 1-1-1 Tennoudai, Tsukuba, Ibaraki 305-8577, Japan

**Keywords:** Diatoms, Differential Gene Loss, EF-1α, EFL, Functional Remodeling, *Goniomonas*, *Pythium*, *Spizellomyces*, *Thecamonas*

## Abstract

**Background:**

Elongation factor-1α (EF-1α) and elongation factor-like (EFL) proteins are functionally homologous to one another, and are core components of the eukaryotic translation machinery. The patchy distribution of the two elongation factor types across global eukaryotic phylogeny is suggestive of a ‘differential loss’ hypothesis that assumes that EF-1α and EFL were present in the most recent common ancestor of eukaryotes followed by independent differential losses of one of the two factors in the descendant lineages. To date, however, just one diatom and one fungus have been found to have both EF-1α and EFL (dual-EF-containing species).

**Results:**

In this study, we characterized 35 new EF-1α/EFL sequences from phylogenetically diverse eukaryotes. In so doing we identified 11 previously unreported dual-EF-containing species from diverse eukaryote groups including the Stramenopiles, Apusomonadida, Goniomonadida, and Fungi. Phylogenetic analyses suggested vertical inheritance of both genes in each of the dual-EF lineages. In the dual-EF-containing species we identified, the EF-1α genes appeared to be highly divergent in sequence and suppressed at the transcriptional level compared to the co-occurring EFL genes.

**Conclusions:**

According to the known EF-1α/EFL distribution, the differential loss process should have occurred independently in diverse eukaryotic lineages, and more dual-EF-containing species remain unidentified. We predict that dual-EF-containing species retain the divergent EF-1α homologues only for a sub-set of the original functions. As the dual-EF-containing species are distantly related to each other, we propose that independent re-modelling of EF-1α function took place in multiple branches in the tree of eukaryotes.

## Background

Elongation factor 1α (EF-1α) proteins in eukaryotes and archaebacteria, and their orthologues in bacteria (elongation factor Tu), are GTPases required for the central process of translation [1,2]. The primary sequence of EF-1α is highly conserved across the tree of life, suggesting that this protein was established in the last universal common ancestor, and inherited by extant organisms [3]. However, genomic and transcriptomic data from diverse organisms have shown that some eukaryotic lineages lack EF-1α, and these lineages instead were found to possess a putative EF-1α-related GTPase [4]. These elongation factor-like (EFL) proteins are believed to perform the same function in translation as EF-1α, as there is no significant functional divergence in the regions that are critical for EF-1α function [4]. The functional equivalence of EFL and EF-1α would explain the mutually exclusive distributions of EFL and EF-1α genes amongst eukaryotes since EF-1α would be functionally redundant in eukaryotes with EFL-mediated translation elongation, and *vice versa*.

Intensive surveys for EFL genes in phylogenetically diverse eukaryotes revealed a number of groups that have both ‘EF-1α-containing’ and ‘EFL-containing’ species [5-10]. The co-existence of EF-1α-containing and EFL-containing species in a monophyletic group can be explained by the ancestral co-occurrence of EF-1α and EFL, and subsequent losses of either of the two elongation factors in the descendants. Henceforth, we designate the above scenario simply as the ‘differential loss’ hypothesis [8]. Many aspects of this hypothesis are difficult to test experimentally. Nonetheless, dual expression of EF-1α and EFL proteins in *Trypanosoma brucei* cells, which corresponds to the ancestral state assumed in the differential loss hypothesis, had no apparent impact on cell viability [11].

It was previously found that examined diatom species were either EF-1α-containing or EFL-containing, except for a single species, *Thalassiosira pseudonana*, whose genome encodes both EF-1α and EFL genes [7]. According to the differential loss hypothesis described above, the EF-1α/EFL gene data from diatoms can be explained as follows: (1) the ancestral diatom genome was ‘dual-EF-containing,’ (2) the *T. pseudonana* genome retains the ancestral state, and (3) the EF-1α (or EFL) gene was lost in the extant EFL-containing (or EF-1α-containing) descendants [7]. A similar situation has been proposed for Fungi; although the vast majority of fungal species are either EF-1α-containing or EFL-containing, a single species, *Basidiobolus ranarum,* was found to be dual-EF-containing [12]. Under the differential loss hypothesis, *T. pseudonana* and *B. ranarum* retain the ancestral state of diatom and fungal genomes, respectively.

The differential loss hypothesis is an increasingly popular explanation of the current EF-1α/EFL gene distribution in the tree of eukaryotes. Nevertheless, dual-EF-containing species, which are believed to reflect the ancestral state of their phylogenetic relatives containing either EF-1α or EFL, have, to date, only been described in diatoms and Fungi. In this study, by experimental surveys and data mining in publicly available genome and/or transcriptomic data, four independent lineages—Stramenopiles, Apusomonadida, Goniomonadida, and Fungi—were found to contain at least one dual EF-containing species (11 species were newly identified in total). All EF-1α genes in the dual EF-containing species examined here appear to be divergent, and are transcribed at a much lower level than the co-occurring EFL genes, suggesting that EF-1α has functionally diverged in these species. We propose that the re-modeling of the original EF-1α functions seemingly occurred in several independent branches of the tree of eukaryotes.

## Results

**Figure 1:**
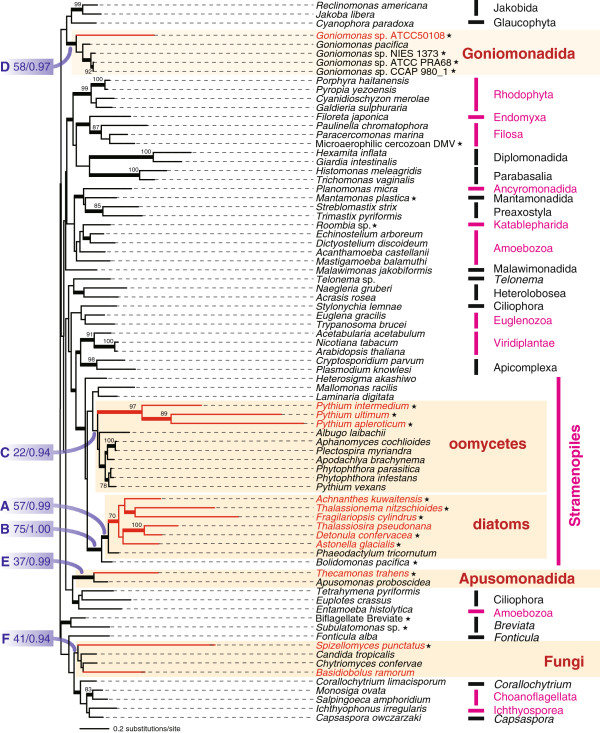
**EF-1α phylogeny.** The unrooted maximum-likelihood tree was inferred from 79 EF-1α sequences with 400 unambiguously aligned amino acid positions. Bootstrap values less than 70% are not shown except at nodes that are relevant to EF-1α gene evolution in Fungi, diatoms, oomycetes, and Apusomonadida (nodes **A** to **F**). The nodes supported by Bayesian posterior probabilities ≥ 0.95 are highlighted by thick lines. Branches leading to the taxa containing both EFL and EF-1α genes are highlighted in red. The lineages comprising both EF-1α-containing and EFL-containing species are highlighted in magenta. The new sequences isolated/identified in this study are indicated by stars.

**Figure 2:**
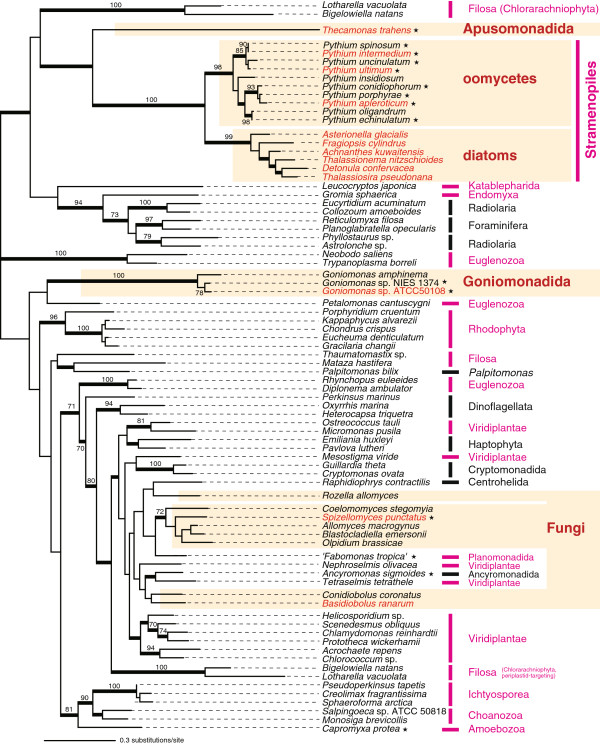
**EFL phylogeny.** The unrooted maximum-likelihood tree was inferred from 80 EFL sequences with 407 amino acid positions. Only bootstrap values ≥ 70% are shown. The nodes supported by Bayesian posterior probabilities ≥ 0.95 are highlighted by thick lines. All other details of the figure are as described in the legend to Figure 1.

We successfully isolated/identified 20 and 15 previously unidentified EF-1α and EFL sequences, respectively, by a PCR survey or mining publicly available and in-house genomic/transcriptomic databases (Table 1). Five diatoms, three oomycetes, one goniomonad, one apusomonad, and a chytridiomycete fungus were found to be dual-EF-containing in this study, in addition to the two previously reported dual-EF-containing species, the diatom *T. pseudonana*[7] and a fungus of uncertain taxonomic affiliation, *B. ranarum*[12]. We updated EF-1α and EFL alignments by adding the new sequences listed in Table 1, and both alignments were analyzed with maximum-likelihood (ML) and Bayesian phylogenetic methods (Figures 1 and 2).

**Table 1:** EF-1α and EFL homologues isolated/identified in this study

**Gene**	**Taxon name**	**Classification**	**Survey**	**Accession nos.**
EF-1α*	*Detonula confervcace* CCMP353	Diatoms	PCR	AB766031
EF-1α*	*Achnanthes kuwaitensis* NIES1349	Diatoms	PCR	AB775895
EF-1α*	*Fragilariopsis cylindrus*	Diatoms	Genome, public	See Additional file 3
EF-1α*	*Thalassionema nitzschioides* NIES534	Diatoms	PCR	AB766032
EF-1α*	*Asterionella glacialis* NIES417	Diatoms	PCR	AB766030
EF-1α	*Bolidomonas pacifica* CCMP1866	Bolidophyceae	PCR	AB766033
EF-1α*	*Pythium intermedium* MAFF306022	Oomycetes	PCR	AB766039
EF-1α*	*Pythium ultimum* DAOM BR144	Oomycetes	Genome, public	See Additional file 3
			EST, public	
EF-1α*	*Pythium apleroticum* MAFF425515	Oomycetes	PCR	AB766038
EF-1α	*Goniomonas* sp. ATCC PRA68	Goniomonadida	PCR	AB766034
EF-1α*	*Goniomonas* sp. ATCC 50180	Goniomonadida	PCR	AB766037
EF-1α	*Goniomonas truncata* NIES 1373	Goniomonadida	PCR	AB766036
EF-1α	*Goniomonas* sp. CCAP 980_1	Goniomonadida	PCR	AB766035
EF-1α*	*Spizellomyces punctatus* DAOM BR117	Chytridiomycota	Genome, public	See Additional file 3
			EST, public	
EF-1α	*Subulatomonas* sp. strain PCMinv5	*Breviata*	EST, in-house	AB766043
EF-1α	Breviata-like biflagellate strain PCbi66	*Breviata*	EST, in-house	AB766042
EF-1α	*Roombia* sp. NY0200	Katablepharida	PCR	AB766040
EF-1α	*Mantamonas plastica* Bass1 (CCAP 1946/1)	Mantamonadida	EST, in-house	AB766041
EF-1α*	*Thecamonas trahens* ATCC50062	Apusomonadida	EST, public	See Additional file 3
			Genome, public	
EF-1α	microaerophilic cercozoan strain DMV	Filosa	EST, in-house	AB824019
EFL^¶^	*Goniomonas* sp. ATCC 50180	Goniomonadida	PCR	AB766045
EFL	*Goniomonas* sp. NIES 1374	Goniomonadida	PCR	AB766044
EFL	*Pythium spinosum* MAFF425453	Oomycetes	PCR	AB766051
EFL^¶^	*Pythium intermedium* MAFF306022	Oomycetes	PCR	AB766049
EFL	*Pythium uncinulatum* MAFF240295	Oomycetes	PCR	AB766052
EFL	*Pythium conidiophorum* MAFF245320	Oomycetes	PCR	AB766047
EFL	*Pythium porphyrae* MAFF239483	Oomycetes	PCR	AB766050
EFL^¶^	*Pythium apleroticum* MAFF425515	Oomycetes	PCR	AB766046
EFL	*Pythium echinulatum* MAFF425510	Oomycetes	PCR	AB766048
EFL	*Pythium insidiosum* CBS119452	Oomycetes	EST, public	See Additional file 3
EFL^¶^	*Spizellomyces punctatus* DAOM BR117	Chytridiomycota	Genome, public	See Additional file 3
			EST, public	
EFL^¶^	*Thecamonas trahens* ATCC50062	Apusomonadida	Genome, public	See Additional file 3
			EST, public	
EFL	*Capromyxa protea* CF08-5 (ATCC PRA-324)	Tubulinida	EST, in-house	AB766053
EFL	*Fabomonas tropica* strain NYK3C	Ancyromonadida	EST, in-house	AB766055
EFL	*Ancyromonas sigmides* strain B70 (CCAP 1958/3)	Ancyromonadida	EST, in-house	AB766054

*co-occurred with EFL, ^¶^co-occurred with EF-1α. Accession numbers for the sequences obtained by public database search are not described, but their protein sequences were shown in Additional file 3.

### Dual-EF-containing species in diatoms

The majority of diatom species, in which EF-1α/EFL sequences have been characterized to date, appear to possess EFL genes, except for the genomes of *Phaeodactylum tricornutum*[13], which encodes only an EF-1α gene, and *T. pseudonana,* which encodes both EF-1α and EFL genes [7]. In this study, we surveyed EF-1α/EFL genes in diatoms further, and identified five more dual-EF-containing species, indicating that dual-EF-containing species are quite prevalent amongst diatoms. EF-1α transcripts were detected in *Detonula confervacea*, *Achnanthes kuwaitensis*, *Fragilariopsis cylindrus*, *Thalassionema nitzschioides*, and *Asterionella glacialis*, all of which were previously considered to be ‘EFL-containing’. In the EF-1α ML tree, all diatom homologues grouped together with an ML bootstrap value (MLBP) of 57% (node A in Figure 1), and this group branches with the EF-1α homologues of the bolidophyte *Bolidomonas pacifica*. Although the statistical support for the diatom-*Bolidomonas* affiliation was moderate (MLBP = 75%; node B in Figure 1), this particular affiliation found in the EF-1α phylogeny is consistent with their close (organismal) relationships [14]. Thus we concluded that there had been vertical descent of EF-1α genes in the diatom-*Bolidomonas* clade. As shown in previous studies e.g., [7], the updated EFL phylogeny also includes a diatom clade, indicating the vertical descent of EFL genes in this lineage (Figure 2).

Quantitative reverse transcriptase PCR (qRT-PCR) assays revealed that the expression level of the EFL gene is much greater than that of the EF-1α gene in each of the dual EF-containing diatom species identified in this study (Table 2), except for *F. cylindrus,* for which these assays were not performed. However, EF-1α transcripts are likely much less abundant than EFL transcripts in *F. cylindrus* as well, since only EFL transcripts were detected in the *F. cylindrus* transcriptomic data publicly available from the Joint Genome Institute (http://genome.jgi.doe.gov/).

**Table 2:** Relative copy numbers of EF-1α and EFL transcripts by qRT PCR

**Organism**	**EFL**	**(sd)**	**EF-1α**	**(sd)**
*Goniomonas* sp. ATCC 50108	1.50	(0.28)	4.10 × 10^-4^	(7.63 × 10^-5^)
*Asterionella glacialis*	38.77	(6.29)	0.09	(0.04)
*Achnanthes kuwaitensis*	23.22	(1.52)	0.01	(0.002)
*Detonula confervacea*	0.08	(0.03)	4.88 × 10^-4^	(0.11 × 10^-4^)
*Thalassionema nitzschiodes*	0.33	(0.10)	6.36 × 10^-4^	(9.55 × 10^-4^)

Notes—normalized by the copy number of α-tubulin transcripts.

### Dual-EF-containing species in oomycetes

Only EF-1α homologues were identified in well-studied members of the Oomycetes (e.g., *Phytophthora infestans,* for which a complete genome is available [15]), but some of us have recently reported EFL genes in *Pythium oligandrum* and *Pythium ultimum*[16]. In this study, we resurveyed EF-1α/EFL sequences in 8 members of the genus *Pythium*, and identified *Pythium intermedium*, *Py. ultimum*, and *Py. apleroticum* as dual-EF-containing. The EF-1α phylogenetic analysis successfully recovered the monophyly of all of oomycetes, suggesting that *Py. intermedium*, *Py. ultimum*, and *Py. apleroticum* vertically inherited their EF-1α genes from a common oomycete ancestor. We suspect that the ML bootstrap support for the oomycete clade in the EF-1α analysis was lowered due to the divergent nature of the *Py. intermedium*, *Py. ultimum*, and *Py. apleroticum* homologues (MLBP = 22%; node C in Figure 1). The EFL phylogeny also robustly unites all oomycete EFL sequences, including those of the three dual-EF-containing *Pythium* spp. (Figure 2).

The EF-1α gene of *Py. ultimum* is seemingly much less transcribed than its EFL gene. In Illumina transcriptomic data, the k-mer frequency for EFL contig was significantly higher than that for a cytoskeletal protein, α-tubulin (Table 3). In sharp contrast, no contig for EF-1α was obtained in the transcriptomic data (Table 3), even though our RT-PCR successfully detected EF-1α transcripts in *Py. ultimum* (data not shown).

**Table 3:** k-mer frequencies for EF-1α, EFL, and α-tubulin in transcriptomic data

		**k-mer frequency**		
**Organisms**	**Data sources**	**EFL**	**EF-1α**	**α-tubulin**
*Thecamonas trahens*	SRR343042	1540	21	530
*Spizellomyces punctatus*	SRR343043	4797	7	805
*Pythium ultimum*	SRR059026	556	Not detected	31

### Dual-EF-containing species in goniomonads

Prior to this study, EF-1α/EFL data were available for only two goniomonad species: EF-1α transcripts were detected in *Goniomonas pacifica*[17], while an EFL gene was isolated from *Goniomonas amphinema*[18]. In this study, we experimentally surveyed EF-1α/EFL sequences in five *Goniomonas* strains (ATCC 50108, ATCC PRA68, NIES-1373, NIES-1374, and CCAP 980_1). Of these, strain ATCC 50108 appeared to be dual-EF-containing (Table 1). A qRT-PCR assay revealed that EFL transcripts were more abundant than EF-1α transcripts in strain ATCC 50108 (Table 2).

The EF-1α sequences amplified from strains ATCC 50108, ATCC PRA68, NIES-1373, and CCAP 980_1, together with that of *G. pacifica*, formed a clade in the EF-1α phylogeny (MLBP = 58%; node D in Figure 1). The new EFL homologues from strains NIES 1374 and ATCC 50108 showed a close relationship to the *G. amphinema* homologue (Figure 2). Both EF-1α and EFL phylogenies suggest vertical inheritance of the genes encoding the two elongation factors in this lineage.

### Other dual-EF-containing species in Apusomonadida and Fungi

We detected both EFL and EF-1α sequences in both whole-genome shotgun and transcriptomic data from the apusomonad *Thecamonas trahens* (http://www.broadinstitute.org/). The EF-1α sequences of two apusomonads, *T. trahens* and *Apusomonas proboscidea*, grouped together in the ML tree topology (MLBP = 37%; node E in Figure 1), consistent with their organismal relationship. The large discrepancy in branch length between the two apusomonad sequences is likely responsible for the low ML bootstrap support. In the EFL phylogeny, the *T. trahens* sequence branched at the base of the diatom-oomycete clade (Figure 2). Unfortunately, the current analysis does not allow us to determine if EFL genes were the result of descent through vertical inheritance in apusomonads, because: (i) only one EFL sequence is known for apusomonads, and (ii) *T. trahens* and opisthokonts were distant from each other in the EFL phylogeny, in contrast to the close organismal relationship between apusomonads and opisthokonts e.g., [19].

Our EF-1α/EFL gene survey also identified the genome of the chytridiomycote fungus *Spizellomyces punctatus* as encoding both kinds of elongation factors. The EF-1α sequences of *S. punctatus* and *B. ranarum* bore the well-known Opisthokonta-specific insertion (Additional file 1), and formed a clade with other fungal sequences in the phylogenetic analyses (MLBP = 41%; node F in Figure 1), suggesting that the EF-1α genes of *S. punctatus* and *B. ranarum* and those of other fungal species share an exclusive ancestry. Again, the grouping of the two long-branched sequences of *S. punctatus* and *B. ranarum* with other fungal sequences did not receive high ML bootstrap support. We are currently unsure whether the extant EFL genes in fungi are the descendents of a single gene in the ancestral fungal species: The monophyly of fungi was not recovered in the ML tree inferred from the EFL alignment (Figure 2), but the approximately unbiased test [20] failed to reject the alternative hypothesis, in which all fungal EFL sequences were enforced to be monophyletic, at the 5% level (data not shown).

In both *T. trahens* and *S. punctatus* there is a large difference in transcriptional levels between EF-1α and EFL genes. In the transcriptomic data of the two species, the k-mer frequency for EFL was much greater than that for EF-1α (Table 3), as seen in other dual-EF-containing species (see above).

### New EF-1α/EFL data from other eukaryotes

Our EF-1α/EFL survey successfully revealed that the taxa Katablepharida, Amoebozoa (or a subgroup of Amoebozoa), and Ancyromonadida contain both EFL-containing and EF-1α-containing species. For amoebozoans and ancyromonads, only EF-1α-containing species were known prior to this study (see Figure 1), however, we detected EFL sequences in the amoebozoan *Copromyxa protea* and the ancyromonad *Fabomonas tropica* (Table 1). Likewise, the first-surveyed katablepharid *Leucocryptos marina* was EFL-containing [16], but a RT-PCR survey of a secondly-surveyed katablepharid, *Roombia* sp., identified EF-1α transcripts (Table 1).

## Discussion

### Several eukaryote lineages include multiple dual-EF-containing species

**Figure 3:**
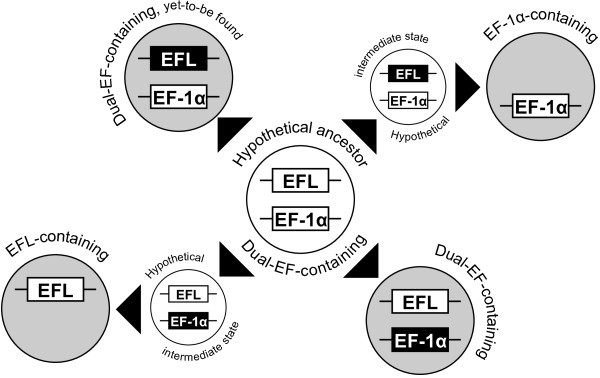
**Scheme for EF-1α/EFL evolution in eukaryotes.** A differential loss process from the hypothetical dual-EF-containing ancestor (center; open) produced four descendent types (shaded): (i) EFL-containing descendent (lower left), (ii) EF-1α-containing descendent (upper right), (iii) dual-EF-containing descendent with a transcriptionally suppressed EF-1α (lower right), and (iv) dual-EF-containing descendent with a transcriptionally suppressed EFL gene (upper left). The EF-1α gene is blackened in the descendent shown in lower right, as this gene is functionally reduced and transcriptionally suppressed, which is likely analogous to the hypothetical intermediate that leads to the EFL-containing type that lacks EF-1α. Likewise, the other type of dual-EF-containing descendent (upper left), if exist, bears the re-modeled EFL gene (blackened), and is analogous to the hypothetical intermediate that led to the EF-1α-containing descendants that lack EFL.

Ancestral co-occurrence of EF-1α and EFL followed by differential loss of one of the two elongation factors most likely shaped the current EF-1α/EFL distribution within eukaryotes. In this scenario, the extant dual-EF-containing species retain the ancestral state and thus are analogous to the inferred intermediates that led to descendant lineages that contain either EF-1α or EFL (Figure 3). In this study, we found 11 new dual-EF-containing species in four distantly related lineages: (1) Goniomonadida, (2) Apusomonadida, (3) Stramenopiles (including diatoms and oomycetes), and (4) Fungi (including *S. punctatus* and *B. ranarum*). In light of the differential loss process proposed for EF-1α/EFL evolution, we speculate that more dual-EF-containing species remain undetected in other lineages that contain both EF-1α-containing and EFL-containing species, including: Viridiplantae [6], Euglenozoa [8], Choanoflagellata [5], Endomyxa [10], Filosa [9], Rhodophyta [18], Katablepharida (this study), Amoebozoa (this study), and Ancyromonadida (this study) (highlighted in pink in Figures 1 and 2). Considering the revised distribution of EF-1α/EFL genes, we cannot exclude the possibility that the last eukaryotic common ancestor was dual-EF-containing.

Finally, it will be of interest to continue surveying dual-EF-containing species, especially within Stramenopiles and Fungi. Kamikawa et al. [16] postulated that the dual-EF status can be traced back to the ancestral stramenopile species, based on the monophyly of stramenopiles in EF-1α phylogenies (Figure 1), and of diatoms and oomycetes in EFL phylogenies (Figure 2: Note that no EFL homologue has been identified to date in any stramenopile subgroups except diatoms and oomycetes). Thus, we predict that dual-EF-containing species should be found in so-far unsampled stramenopiles. Similarly, *S. punctatus* and *B. ranarum* are unlikely to be the sole fungal species with a dual-EF status, given that the most recent common ancestral fungus was proposed to be dual-EF-containing [12].

### Parallel re-modeling of EF-1α function in eukaryotic evolution

In the dual EF-containing diatom *T. pseudonana*, some of us [7] proposed that the EF-1α homolog performs only a subset of its original functions, and does not participate in protein synthesis as an elongation factor, for the following reasons. Firstly, in an EF-1α phylogeny, the *T. pseudonana* homologue was much more divergent than that of a closely related EF-1α-containing species, *P. tricornutum*, suggesting that the former is under fewer functional constraints than the latter. Secondly, EF-1α transcripts were much less abundant in *T. pseudonana* than the transcripts of EFL or of an α-tubulin gene. As observed in *T. pseudonana*, the five dual EF-containing diatoms identified in this study (i.e. *A. kuwaitensis*, *A. glacialis*, *D. confervacea*, *F. cylindrus*, and *T. nitzschioides*) appeared to possess divergent EF-1α genes (Figure 1). In each of the five diatoms, the transcriptional level of the EF-1α gene was heavily suppressed compared to that of the co-occurring EFL gene (Table 2). Thus, the five dual-EF-containing diatoms most likely use EFL as the principal elongation factor, while a sub-set of the original EF-1α functions is assigned to the divergent EF-1α. These dual-EF-containing diatoms have most likely re-modeled their EF-1α functions, such that they carry out only the auxiliary roles that the proteins originally performed, such as interactions with cytoskeletal proteins and ubiquitin-dependent protein degradation [1,21,22].

It is likely that similar re-modeling of EF-1α function has also occurred in other dual-EF-containing lineages. In the non-diatom dual-EF-containing species, the EF-1α sequences were also divergent (Figure 1), and were transcribed at a low level compared to the co-occurring EFL genes (Tables 2 and 3). These results strongly suggest that dual-EF-containing species in general utilize EF-1α for subsets of the original functions, while EFL participates in translation as a core factor. Significantly, the re-modeling of EF-1α function probably took place separately in Stramenopiles (including diatoms and oomycetes), Goniomonadida, Apusomonadida, and Fungi, as these lineages are distantly related to one another in the organismal phylogeny. Moreover, diatoms (photosynthetic heterokont algae) and oomycetes (non-photosynthetic stramenopiles) may have also re-modeled their EF-1α functions in parallel as they are relatively distantly related within stramenopiles. We also suspect that parallel re-modeling of EF-1α function occurred within Fungi, as *S. punctatus* and *B. ranarum* are not particularly close relatives [12].

We are currently unsure about the precise functions of the divergent EF-1α in the dual-EF-containing species. Under the parallel re-modeling scenario proposed above, the suite of retained EF-1α functions could vary between any of two dual-EF-containing lineages. However, the overall substitution patterns in divergent EF-1α sequences in distantly related dual-EF-containing species are found to be similar to each other (Additional file 2). This observation hints at parallel loss of the same aspects of EF-1α function and retention of a subset of original functions in multiple dual-EF-containing lineages scattered over the tree of eukaryotes. These speculations could be tested more directly by biochemical studies of EF-1α function in selected representatives of these lineages.

## Conclusions

According to the differential loss hypothesis for EF-1α/EFL evolution, a dual-EF-containing ancestor likely gave rise to two types of descendants—one containing only EFL and the other containing only EF-1α. Nevertheless, EF-1α/EFL surveys, including this study, have identified an additional type of descendent retaining the ancestral arrangement (i.e. dual-EF-containing) in multiple branches of the tree of eukaryotes. If EF-1α/EFL sequences are surveyed in a broader spectrum of eukaryotes, it is highly likely that the number and diversity of known dual-EF-containing species will grow further.

Curiously, all dual-EF-containing species identified so far appear to retain divergent, low-expressed EF-1α genes (see above), which are analogous to the hypothetical intermediate leading to EFL-containing descendants (Figure 3). We suspect that the multiple functions of the canonical EF-1α may have prevented the dual-EF-containing cells from losing this protein immediately after EFL took over from EF-1α as the core translation factor. The presence of dual-EF-containing species indicates that the adoption of EFL as the dominant core factor in translation does not necessarily lead to the elimination of EF-1α from the entire cellular system.

Curiously, we found little evidence for living analogues of the hypothetical intermediate that led to EF-1α-containing descendants, which would possess a divergent, low-transcribed EFL gene. The presence or absence of dual-EF-containing species, in which a divergent EFL gene is transcribed at lower levels than the co-occurring EF-1α gene, would be crucial to understanding the evolutionary processes that shaped the current EF-1α/EFL gene distribution across the tree of eukaryotes. We need to re-examine EFL sequences in the species currently recognized as ‘EF-1α-containing’ since low-expressed EFL genes might be overlooked in these taxa, especially if genomic or high-coverage transcriptomic data is lacking.

## Methods

### Strains

*Achnanthes kuwaitensis* (NIES-1349), *Asterionella glacialis* (NIES-417), *Thalassionema nitzschioides* (NIES-534), *Goniomonas amphinema* (NIES1371), *Goniomonas truncata* (NIES-1373), and *Goniomonas* sp. (NIES-1374) were purchased from the Microbial Culture Collection at the National Institute for Environmental Study in Japan. *Detonula confervacea* (CCMP353) and *Bolidomonas pacifica* (CCMP1866) were purchased from the Provasoli-Guillard National Center for Marine Algae and Microbiota. *Goniomonas* sp. (CCAP 980/1) was purchased from the Culture Collection of Algae and Protozoa. *Goniomonas* sp. (ATCC PRA-68) and *Goniomonas* sp. (ATCC 50108) were purchased from American Type Culture Collection. *Pythium apleroticum* (MAFF425515), *Py. conidiophorum* (MAFF245320), *Py. echinulatum* (MAFF425510), *Py. intermedium* (MAFF306022), *Py. porphyrae* (MAFF239483), *Py. spinosum* (MAFF425453), *Py. ultimum* (MAFF425505), and *Py. uncinulatum* (MAFF240295) were purchased from the GeneBank (Microorganism Section) at the National Institute of Agrobiological Sciences in Japan. *Roombia* sp. strain NY0200 was cultivated with bacterial prey in URO-YT medium (Moriya et al. 2000). RNA was extracted from the harvested cells by using an RNeasy Plant Mini kit (QIAGEN), and then subjected to oligo(dT)-primed reverse transcriptase (RT) reactions by using the 3’ rapid amplification of cDNA ends kit (Invitrogen). Each of the two procedures described above was conducted following the corresponding manufacturers’ instructions.

### PCR-based survey of EF-1α and EFL transcripts

We amplified EF-1α and/or EFL sequences of *Roombia* sp., diatoms, *Bolidomonas pacifica*, and goniomonads (see the previous section) by a two-step procedure: For the first RT-PCR, the combination of one of three forward primers (5′-GGCCACGTGGAYTCNGGNAARTCNAC, 5′-GGCCACGTGGAYAGYGGNAARTCNAC, or 5′-GGCCACGTGGAYGCNGGNAARTCNAC) and a reverse primer (5′-ACGAAATCTCTCTTRTGNCCNGGNGCRTC) were used. These primer sets can amplify the 5′ portions of the transcripts (~250 bp in length) for EF-1α and EFL, as well as other EF-1α-related proteins in a single reaction. For each reaction, amplicons were cloned into pGEMTEasy vector (Promega), and sequenced ≥12 clones to survey EF-1α/EFL sequences. Secondly, the 3′ portions of *Roombia*, diatom, and goniomonad EF-1α/EFL transcripts were amplified by the 3′ rapid amplification of cDNA ends (RACE) kit (Invitrogen) with exact-match primers based on the nucleotide sequences of the initial amplicons. We amplified the 3′ portion of the EF-1α transcript of *B. pacifica* by the combination of an exact-match primer (see above) and a degenerate primer, which can anneal to the 3′ portion of EF-1α open reading frame (5′-CAGAATTGCGACAGCNACNGTYTG). Amplicons were cloned and sequenced completely as described above.

From all of the seven species belonging to the oomycete genus *Pythium* examined in this study, we obtained the amplicons covering most of the EFL-coding region by RT-PCR with a set of primers 5′-AGCCGAGAAGGGTGGTTTCG and 5′-ACAGATAATCTGACCAACACC. The details of cloning and sequencing of the EFL amplicons were same as described above.

We then screened the 5′ portion of EF-1α sequences in the seven *Pythium* spp. in two separate trials. Firstly, we applied the combinations of primers for EF-1α sequences in phylogenetically diverse eukaryotes; two forward primers (5′-GTGGACGCCGGNAARTCNACNACNAC and 5′-GTGGACGCCGGNAARAGYACNACNAC) and two reverse primers (5′-TCGGCCTGGGANGTNCCNGTNATCAT and 5′-TCGGCCTGGGTNGTNCCNGTNATCAT). The RT-PCR with these ‘universal’ primers succeeded in amplifying the partial EF-1α transcripts in *Py. apleroticum*. For the second trial, we prepared new degenerate primers, which were more specific to oomycete EF-1α sequences than those used in the first trial: PytEF1aFA, PytEF1aFB, and PytEF1aR (5′-TCGGCAAGACGTCGTWCAAGTAC, 5′-GGTCACCGCGATTTCATCAAGAAC, and 5′-GACNGGNACCGTGCCAATACC, respectively). EF-1α transcripts in the *Pythium* spp. were surveyed by the hemi-nested RT-PCR, in which the combination of PytEF1aFA and PytEF1aR, and that of PytEF1aFB and PytEF1aR were used for the first and second reactions, respectively. The partial EF-1α transcript in *Py. intermedium* was amplified in the second trial with the ‘oomycete-oriented’ primers. We could not detect any EF-1α transcripts in the *Pythium* species examined in this study, other than *Py. apleroticum* and *Py. intermedium*. The 3′ portions of *Py. apleroticum* and *Py. intermedium* EF-1α transcripts were amplified by the 3′ RACE, followed by cloning and sequencing. The details of the 3′ RACE, and cloning and sequencing of the amplicons were same as described above.

### Illumina transcriptomic analyses

We obtained transcriptomic data from the following organisms; two ancyromonads, *Ancyromonas sigmides* B70 (CCAP 1958/3) and *Fabomonas tropica* NYK3C, the breviates, *Breviata*-like biflagellate PCbi66 and *Subulatomonas* sp. PCMinv5, the mantamonad *Mantamonas plastica* Bass1 (CCAP 1946/1), the tubulinid amoebozoan *Capromyxa protea* CF08-5 (ATCC PRA-324), and the microaerophilic cercozoan strain DMV.

*A. sigmoides* and ‘*F. tropica*’ were cultivated with bacterial prey (*Enterobacter aerogenes*) in a mixture of 50% ATCC 802 medium and 50% filtered sterile seawater, and in a mixture of 50% seawater and 50% ddH_2_O, respectively. Strain PCbi66 was grown in ATCC 1525 medium with bacterial prey (*Klebsiella pneumoniae* ATCC 23432). *Subulatomonas* sp. was cultivated with bacterial prey in ATCC 1773 medium made with 50% seawater and 50% ddH_2_O. *M. plastica* was grown with bacterial prey (*K. pneumoniae* ATCC 23432) in a mixture of 50% seawater and 50% ddH_2_O. *C. protea* was grown on weak malt yeast agar plates (0.02 g Yeast extract, 0.02 g Malt extract, 0.75 g K_2_HPO_4_, 1 L ddH_2_O, 15 g Agar) with streaks of *Escherichia coli* as food. Stain DMV was grown in ATCC 802 medium, with bacterial prey (*K. pneumoniae* ATCC 23432) killed at 65°C for 1 hour.

Total RNA was isolated using Trizol (Tri-reagent) following the protocol supplied by the manufacturer (Sigma). Construction of cDNA libraries and illumina RNAseq was performed by Macrogen (South Korea) for strain PCbi66 and *A. sigmoides*, by GeneWiz (USA) for ‘*F. tropica*’, *Subulatomonas* sp., and *M. plastica*, and by the Institut de Recherche en Immunologie et Cancérologie (IRIC) of Universite de Montreal (Canada) for *C. protea* and strain DMV.

Raw sequence read data were filtered based on quality scores with the fastq_quality_filter program of FASTXTOOLS (http://hannonlab.cshl.edu/fastx_toolkit/), using a cutoff filter (a minimum 70% of bases must have quality of 20 or greater). Filtered sequences were then assembled into clusters using the Inchworm assembler of the TRINITY r2001-5-13 package [23]. EF-1α/EFL sequences were identified using basic local alignment search tool (tblastn).

### Database search of EFL and EF-1α genes

By using *T. pseudonana* EFL and EF-1α amino acid sequences as the queries, we performed tblastn searches with *E*-value cutoff < 10^-100^. Putative EF-1α/EFL sequences identified by the initial tblastn search were then confirmed by blastp searches with *E*-value cutoff < 10^-100^. The reciprocal similarity searches identified both EFL and EF-1α genes in the genomes of *T. trahens* and *S. punctatus* from the whole genome shotgun database in NCBI (http://www.ncbi.nlm.nih.gov/)*.* Likewise, both EF-1α and EFL genes were detected in the genome databases of the diatom *F. cylindrus* (http://genome.jgi-psf.org/Fracy1/Fracy1.home.html) and the oomycete *Py. ultimum* (http://pythium.plantbiology.msu.edu/). For the Illumina RNAseq data of *T. trahens*, *S. punctatus*, and *Py. ultimum* we collected raw sequence data from the NCBI’s Short Reads Archive (SRA), accessions SRR343042, SRR343043, and SRR059026, respectively. These raw data were assembled into clusters using the Inchworm assembler of the TRINITY r2001-5-13 package, as above. We then identified the contigs pertaining to EFL, EF-1α, and α-tubulin through tblastn, and compared the k-mer frequency of each respective contig to compare the relative transcriptional level between the co-occurring EFL and EF-1α genes (Table 3). We provide the amino acid sequences mentioned here as Additional file 3.

### Phylogenetic analysis

EFL and EF-1α amino acid sequences were sampled from the broad spectrum of eukaryotes. Datasets of the two elongation factor families were separately aligned, and then ambiguously aligned positions were excluded before phylogenetic analyses. The final EFL and EF-1α datasets contained 80 sequences with 407 amino acid positions and 79 sequences with 400 amino acid positions, respectively. The two datasets were analyzed using both ML and Bayesian phylogenetic methods. ML analyses were performed using RAxML 7.2.1 [24] under the LG model [25] incorporating empirical amino acid frequencies and among-site rate variation approximated by a discrete gamma distribution with four categories (LG + Γ + F model). The ML tree was estimated by heuristic searches based on 300 distinct parsimony starting trees. In RAxML bootstrap analyses (1000 replicates), the heuristic tree search was performed from a single parsimony tree per replicate.

The EFL and EF-1α datasets were also subjected to Bayesian analysis using PhyloBayes v.3.3 [26] with the LG + Γ + F model. For the EF-1α analysis, two parallel Markov Chain Monte Carlo (MCMC) runs were run for 63,799 and 63,885 generations, sampling log-likelihoods and every 10 trees (maxdiff = 0.16254; ‘burn-in’ was set as 100 based on the log-likelihood plots). The EFL dataset was analyzed as described above, except two MCMC runs were run for 12,520 and 12,511 generations (maxdiff = 0.113078).

### Quantitative reverse transcriptase (qRT) PCR

To normalize the copy numbers of EFL and EF-1α transcripts, we amplified the α-tubulin sequence of *Goniomonas* sp. ATCC 50108 by RT-PCR with the following degenerate primers: 5′-RGTNGGNAAYGCNTGYTGGGA and 5′-CCATNCCYTCNCCNACRTACCA. To amplify the α-tubulin sequences of diatoms *A. kuwaitensis*, *A. glacialis*, and *T. nitzschioides*, we used a second set of degenerate primers: 5′-GARCTNTAYTGYCTNGARCAYGG and 5′-CGCGCCATNCCYTCNCCNACRTACCA. The α-tubulin sequence of the diatom *D. confervacea* was amplified by using the following primers: 5′-CGCGCCATNCCYTCNCCNACRTACCA and 5′-CGTAGANAGCCTCGTTGTC. The cloning and sequencing of the α-tubulin amplicons were carried out as described above. Accession nos. for the sequences are AB766056 – AB766059.

In Table 4 we list the exact-match primers used for qRT-PCR assays designed based on the EF-1α, EFL, and α-tubulin sequences in the four diatoms and *Goniomonas* sp. ATCC 50108. The plasmids carrying the EFL, EF-1α, and α-tubulin amplicons (see above) were used as the standards for qRT-PCR. A mixture for qRT-PCR contained SYBR Green I (TaKaRa), Premix *ExTaq* (TaKaRa), a set of exact-match primers (final concentration of 0.3 μM each), and template solution: either cDNA, the corresponding RNA sample (the negative control), or five differently diluted plasmid solutions including 10 to 10^7^ copies of the target gene fragments (the standards). The qRT-PCR thermal cycling conditions were 95°C for 30 sec followed by 50 cycles comprised of 95°C for 5 sec, a gene-specific temperature for 10 sec (Table 4), and 72°C for 10 sec. We confirmed that a single target product was amplified by real-time PCR, based on melting curves (data not shown). In each assay, the target amplification from the RNA sample was out of the quantifiable range. Smart Cycler II (Cepheid) and Thermal Cycler Dice (TaKaRa) were used for the assays on the four diatoms and that of *Goniomonas* sp., respectively.

**Table 4:** Primers and annealing temperatures for qRT PCR

**Organisms**	Genes (°C)*	**Primers**
*Achnenthes kuwaitensis*	EFL (57)	5'-GTCACTTGATCTTCAAGCAG
		5'-TGTCGGTGAAGAACTCCTTG
	EF-1α (60)	5'-GAGGAGTTGACGAGAACACG
		5'-TTGGAGACTCGAACTTCCAG
	α-tubulin (60)	5'-TGGAGCCCTACAACTCCATC
		5'-CACCAGGTTGGTCTGGAACTC
*Asterionella glacialis*	EFL (58)	5'-TATCTCTGAGCGTGAGATGAAG
		5'-CTTGGTGTTGCACTGAATGG
	EF-1α (54)	5'-TGAAGAACGAACTATGGAAG
		5'-CCAAAGTGAAATATCGATTG
	α-tubulin (58)	5'-ACATGGCATGCTGCCTCATG
		5'-ATCCTCGAAAGAGCTTCTGC
*Detonula confervacea*	EFL (58)	5'-AGGAATCTCTGCTCGTGAG
		5'-GAACTCCTTGGTGTTACACTG
	EF-1α (58)	5'-GAAACCATCGACAAGTACG
		5'-GAAACTTCCACAACGTGATATCG
	α-tubulin (58)	5'-CAAATGCGCAGCGACAAGAC
		5'-TTCCAGAACGGACCTCGTC
*Thalassionema nitzschioides*	EFL (56)	5'-AATCTCTGCTCGAGAGATGG
		5'-TGTAGTGGTACTTGCCAGTG
	EF-1α (56)	5'-CGTAGCCGAAAGCATAATAG
		5'-CCAGACACTGATATCAATAG
	α-tubulin (56)	5'-TTGTATGATGTCTGCCGTGG
		5'-AAGCCTTCTCACGCGAAATA
*Goniomonas* sp.	EFL (60)	5’-CATCAAGGGTCTCAAGAAGGACAAC
		5’-CAGTTGATGGCGGTCATCTTCATG
	EF-1α (60)	5’-GTTCTCTGCTGGATACACTCCAGTG
		5’-ACGCTATTCATGGAAGGCCTCAAC
	α-tubulin (55)	5’-CATGTACCGTGGTGATGTCG
		5’-CTGGACCTTGGCAAGATCAC

*Numbers in parentheses show primer set-specific annealing temperatures used in qRT PCR.

### Accession numbers

AB766030-AB766059, AB775895, and AB824019.

## Competing interests

Non-financial competing interests.

## Authors’ contributions

RK, MWB, and YN determined sequences. RK, MWB, and YI performed phylogenetic analyses. NY, YS, AH, and RG provided research materials. RK and YI designed the study and wrote the manuscript. MWB, NY, AGBS, AJR, and TH helped to draft the manuscript. All authors read and approved the final manuscript.

## Supplementary Material

Additional file 1 **Partial alignment of EF-1α sequences.** The Opisthokonta-specific insertion is highlighted in grey. Numbers above the alignment are the amino acid positions in *Thalassiosira pseudonana* EF-1α. The divergent EF-1α homologues in the two dual-EF-containing fungi are highlighted by stars. Click here for file

Additional file 2 **Substitution patterns in the divergent EF-1α sequences.** The amino acid sequences of the divergent EF-1α homologues (marked by stars) were compared to those of phylogenetically related, canonical EF-1α proteins. Amino acids are grouped into four Dayhoff categories—(i) acidic residues (D and E), (ii) basic residues (H, K, and R), (iii) polar-uncharged residues (C, N, Q, S, T, W, and Y), and (iv) hydrophobic non-polar residues (A, F, G, I, L, M, P, and V). Substitutions across two out of the four Dayhoff categories between the divergent and canonical EF-1α sequences are highlighted in red. Click here for file

Additional file 3 **EF-1α/EFL sequences identified in publicly available databases.** The amino acid sequences of EF-1α/EFL homologues identified in publicly available databases are listed here. Click here for file
