# Supplementary material for: Parallel re-modeling of EF-1α function: divergent EF-1α genes co-occur with EFL genes in diverse distantly related eukaryotes
Source: BMC Evol Biol. 2013 Jun 26;13:131. doi: 10.1186/1471-2148-13-131 (PMC3699394; doi:10.1186/1471-2148-13-131)
Supplement: Additional file 1 — Partial alignment of EF-1α sequences. The Opisthokonta-specific insertion is highlighted in grey. Numbers above the alignment are the amino acid positions in Thalassiosira pseudonana EF-1α. The divergent EF-1α homologues in the two dual-EF-containing fungi are highlighted by stars. [file 1471-2148-13-131-S1.pdf]

Opisthokonta-specific insertion

```
                   186                                                                      233
                   |                                                                          |
 Corallochytrium   VPISGWVGDNMIEASTN----MDWYKGWE---KDGSV---GGKTLIEALDAVSPPKRPSDKPLRLPL
 Capsaspora        VPISGWHGDN-MEASEN----MPWFKGWTIERKEGNA---SGKTLIEALDAISPPKRPTDKPLRLPL
 Monosiga          VPISGWHGDNMIEASEK----LPWYKGWEITRKDGNA---KGKTLLEALDAIIPPERPTSKPLRLPL
 Ichthyophonus     VPISGWHGDNMVAPTEN----MPWYKGWTCERKEGNT---SGFTLLEALDNIQAPKRPTDKPLRLPL
 Salpingoeca       VPISGWHGDNMIEASEK----LPWYKGWEVQRKDDAGGNAKGKTLLEALDAIHPPQRPTGKPLRLPL
 Fonticula         IPITGFHGDNMIEPTTN----MSWWKGFEITRGSAKL---TGLTLLDALNHIEPPSRPTDKPLRLPL
*Basidiobolus      VPISGFQGDNVFEQSEN----MPWYKGWQRETKAGVS---NGVTLLDAIDSVVPPTRLQDKPLRLPL
 Chytriomyces      VPISGWHGDNMLEASEN----MPRFKGWNKETKAGSS---TGKTLLQAIDAIEPPTRPTDKPLRLPL
 Candida           VPISGWNGDNMIEASTN----CPWYKGWEKETKAGKV---TGKTLLEAIDAIEPPSRPTDKPLRLPL
*Spizellomyces     VPVSGWTGDNLVDPSPN----LPWFSRWKKQTKSGAV---EGKTLLDAIDTIEPPIRPKDKPLRLPI
 Entamoeba         VPISGFQGDNMIEPSTN----MPWY---------------KGPTLIGALDSVTPPERPVDKPLRLPL
 Acanthamoeba      VPISGFHGDNMVDRTDK----MPWY---------------KGPTLLEALDDIKPPKRPMDKPLRVPL
 Giardia           IPTSGWTGDNIMEKSDK----MPWY---------------EGPCLIDAIDGLKAPKRPTDKPLRLPI
 Trichomonas       VPISGWAGDNMTEKSPN----MPWY---------------NGPYLLEALDSLQPPKRPFDKPLRLPL
 Trimastix         VPISGFHGDNMLEPSAN----MPWW---------------KGPTLLGALNNLEVPKRPVDKPLRLPI
 Jakoba            VPISGWNGDNMLERSKN----TPWY---------------TGPTLLEAIDTFSEPKRPHDKPLRCPL
 Reclinomonas      VPISGWNGDNMLERSPN----TPWY---------------KGPIMIEALDLFEEPKRPSDKPLRVPL
 Naegleria         VPISGWTGDNMIEKSDK----MGWY---------------KGPCLLDALDNLIEPVRPTEKPLRLPL
 Euglena           IPISGWNGDNMIEASEN----MGWY---------------KGLTLIGALDNLEPPKRPSDKPLRLPL
 Malawimonas       VPISGWNGDN--EKSPN----MPWY---------------KGFTLLEALDNLEPPKRPFE-PLRLPL
 Cyanophora        VPISGFNGDNMLEPSSN----LGWY---------------KGPTLVEALDQVEEPKRPSEKPLRLPL
 Porphyra          VPTSGWTGENLFERTDKTHALGKWY---------------KGPCLLEALDNCDPPKRPVDKPLRLPL
 Cyanidioschyzon   VPISGWTGDNLFERVPSDHPLAKWY---------------KGPALLEALDAIEPPKRPTEKPLRLPL
 Arabidopsis       VPISGFEGDNMIERSTN----LDWY---------------KGPTLLEALDQINEPKRPSDKPLRLPL
 Nicotiana         VPISGFEGDNMIERSTN----LDWY---------------KGPTLLEALDQINEPKRPSDKPLRLPL
 Acetabularia      VPISGFQGDNMLEKSSN----MNWY---------------KGPTLLEALDMVEPPKRPSDKPLRLPL
 Goniomonas        VPISGWNGDNMIERSPN----ASWY---------------KGPILLEALDQVQAPKRPTDKPLRLPL
 Roombia           VPISGWEGDNMLEKSAR----MPWY---------------KGPTLLEALDNCEEPKRPVDKPLRLPL
 Telonema          IPVSGFHGDNMIEKSTN----MPWY---------------KGDCLLDSLDAAKAPKRPIDKPLRLPL
 Phytophthora      VPISGWEGDNMIDRSTN----MPWY---------------KGPFLLEALDNLNAPKRPSDKPLRLPL
 Laminaria         VPISGWAGDNMVDKSTN----MPWY---------------KGPYLLEALDTMKEPTRPTDKPLRLPL
 Heterosigma       IPISGWNGDSMIEKSPN----MAWY---------------KGPTLLEALDNVNPPKRPTDKPLRLPL
 Bolidomonas       VPISGWSGDNMIDRSPN----MSWY---------------KGPYLLEALDKCNPPKRPTDKALRLPL
 Phaeodactylum     VPISGWEGDNMVEKSTN----MAWY---------------KGPYLLEALDSVTPPKRPTDKALRLPL
 Thalassiosira     VPISGWEGDNITEKSEH----MEWY---------------EGPTLLEALDNVSPPKRPSDKALRIPI
 Pythyum           VPVSGWLGDNLMNRSTS----MPWY---------------DGPTLLEALQQWRPPRRSVDKPLRMLI
 Stylonychia       IPISGWHGDNMLEKSPN----MPWF---------------TGSTLIDALDALDQPKRPKDKPLRLPL
 Tetrahymena       IPISGFNGDNMLERSTN----APWY---------------KGPILVEALDALEPPKRPVDKPLRLPL
 Plasmodium        IPISGFEGDNLIEKSDK----TPWY---------------KGRTLIEALDTMEPPKRPYDKPLRIPL
 Cryptosporidium   VAISGFVGDNMVERSDK----MPWY---------------KGKTLVEALDTMEPPKRPTDKPLRLPL
 Paulinella        IPISGWTGDNMLEKSDK----MPWY---------------KGKCLLEELDSIVPPKRPSGLPLRLPL
 Filoreta          IPISGWNGDNMIEASAN----TPWY---------------KGKTLLDTLDALDPPKRPTDRPLRLPL
 Planomonas        VPISGWSGDNMTEPSAN----MPWY---------------SGPTLLGALDACEVPKRHADKPLRLPL
 Subulatomonas     VPISGWNGDNMLEKSPN----MKWW---------------TGPTLLGALDAVAAPKRPTDKPLRLPL
 Mantamonas        VPISGFHGDNMIEAGDN----MSWY---------------KGPTLLGALDSMEPPTRPVDKPLRLPL
 Thecamonas        VPISGWTGDNMLEPSSN----MSWW---------------TGPTLLEALDSIKAPTRATERPLRIPV
 Apusomonas        VPVSGWNGDNMMEPSPQ----MGWW---------------KGGTLLEALDAITPPARPTDKPLRLPL
```
