# Supplementary material for: Parallel re-modeling of EF-1α function: divergent EF-1α genes co-occur with EFL genes in diverse distantly related eukaryotes
Source: BMC Evol Biol. 2013 Jun 26;13:131. doi: 10.1186/1471-2148-13-131 (PMC3699394; doi:10.1186/1471-2148-13-131)
Supplement: Additional file 3 — EF-1α/EFL sequences identified in publicly available databases. The amino acid sequences of EF-1α/EFL homologues identified in publicly available databases are listed here. [file 1471-2148-13-131-S3.docx]

>Thecamonas trahens EFL

MSAETFDPARKLNVVIIGHVDAGKSTMTGRLIYELGGIDERAKEKLEQAAAENNKAGFAFAYFMDTSKEERERGITIDCQTSSFRTTNYDYSITDAPGHADFITNMITGAGQADVAVLMVPADGNFEASIAKPSGMMAGGQTRQHANLANLLGIKQLVVCVNKMDSSEGGAYNEARFNEIRDEVSRMIDEAGFHSATVPIIPVSGWVGDNLTSPTENMPWYNGWSGRYGYTAKRKKKGFEIVGGTEIKGKTLVEALDNYMRIPDRDANGPLHLSVSGSRLIPGQGLVTMGRIEIGTIRKDDVVALARNGLSGKTFSIQMHHQDVDAGIAGFNVGIVVKFPKGTKPKQVKRGDLLYRPADYADKQELLPRRVKSFRAMIRVQNHPGDLKVGFCPQMCVRTGRCASRISEIHWVMGKRTAGQKLENPEFVRKHEMAEITFEPQGFLYCETFERSESYGRVAGLESKTLVFMGKIIDVEYA

>Pythium insidiosum EFL

MAGEKQHLSLVVCGHVDAGKSTTTGHLIFKLGGIGEREMAKLQAEADAKGKSSFAFAYYMDTCKEERERGVTIQCNTKEFFTDNYHYTIVDAPGHKDYIKNMITGSGCADAGLILVPAEKGGFEAAIAKADPKIGVDEGQTRQHARLLFLLGIEQIVVGVNKMDSCDWSEARYNEIKDEFVKMITQIGFRPKRVPVIPYSGFNGDNLVARTDKAPWYKGWKANLNKDTVIEGYTILDALDKLIKPPKRNPTAPLRIPISNIYNIKGVGQIIC

>Spizellomyces punctatus EFL

MSGEKPHLSIVICGHVDSGKSTTTGRLLFELGGIPEREMEKLRQEAEHLGKSSFAFAFYMDRQKEERARGVTIACTTKEFFTNNYHYTIIDAPGHRDFIKNMISGAAQADVGLLMVPADGNFTTAIQKGDHKAGEIQGQTRQHARLLNLLGVKQLVVGVNKMDSDPAGPYKKERYDEIANEMRNMLVRTGWKKEFVQGSVPVIPISGWIGDNLLKKSEKMSWYQGQEVTALSGKKVKVHTLLDALNDFAEMPERKNDAPMRVPISGIYKIKGVGDVLAGRVEQGTVLPNKDVVFVPTHTAANPCGGKVFTVEMHHKKVEKGLAGDNIGMNIKGLDKNNMPRAGDIMIYKEDKSLKPVKAFTAQVQTLDIPGEVKPGYSPIGFVRCGRSACRMKEIKWKMGKETGMKKLEGAHSLKANEAAEVVFEPIQPLVVDSFKNCEGLSRIAFLDGNSAVMLGKVRDCIVCYECLPSNLILVRTIGNASRIQGLSLLGRIECSFSIVFVI

>Thecamonas trahens EF-1alpha

VIGHVDSGKSTTTGHLIYKCGGIDARQIAKFEQEAGEAGKGSFKYAWVLDKLKSERERGITIDISLWKFETNNYYVTVIDAPGHRDFIKNMITGTSQADCAVLVIDGTRGGFEAGISKDGQTREHALLAYTLGVKQLIVAVNKMDADSVQFSQQRFEEIVAEVKSAIKKVGYNPKKVTFVPISGWTGDNMLEPSSNMSWWTGPTLLEALDSIKAPTRATERPLRIPVQDVYVIDGLGTVPVGRVEAGVIQPGMAIQFAPSGAVAECKSVEMHHEQLEAGNAGDSIGFNVKSLGISDIERGFVASNANDNPAQEVGSFVAQIIVLNHPLFISTGYMPMIDCHTAHIVCRFDELISKLDKRTGKVVEESPRSIAVGDAALVRLVPLMPMCVEPFTAFPPLGRFAVRDMGRTVAVGVIKSVEIKEQAPSGKKGKKKGKGGKGGKRK

>Fragilariopsis cylindrus EF-1alpha

MSTEKVHINLAVIGHVDSGKSTTTDHLLYKCGGIDQQSIEKFETETKEKNGKNGVLDNLPTEREHCTSTTTTTTTTTTTTDISHKNFESSEYQFTIIDTPGHRNYIENMITGISQADVAILIIDSSQYGRYDDEAGGGGICSSGNEDALLAYTFGVKQMIVVINKMDDDTVDFSEERYKSIRYDVSSLLKQVGYKPMKIPFIPISGLLGDNLISKSIHMLWYNGPSLLEALDNVNPPKRRSDKPLRIPIQDVYKINDIQTIATGRVETGVLKPGMKVEFSPSGIINTIETIEINNKTVSQATPGDIVGFTVNNAMNDTDLLLRRGYVTSNSDDQPAQEVSSFEAQVIIMNHPGEITNEYCPIIDCHTSHVPCVFANIKEKMDRRTGKCIEMNPKSVKTGDAFIVELVPKKPLCVEPFTKFPTLGRFVVRDLQQIVAVGVVKSVAYGKVKECE

>Pythium ultimum EF-1alpha

MSVDKARVSFAVIGQIDSGKSTIAGHLLYECGVISDRVLDKLRQEAADMGKASFAYAWVMDNVKSERERGITIEVTMKEFQTPTHVFTAIDTPGHRDFIRNMITGTTQADAALLVTSADPDAHNGEAAQFGNAHEHALLAFMLGVKQVVVAVNKMDHESVMYSRARYEEVKSKVAGYLINIGFKSSLVQFVPVSGWLGDNLMNRSTSMPWYDGPTLLEALQQWRPPRRSVDKPLRMLIDNFYKIRGVGMVITGRVETGVLTPGMTISIAPKGLTAQVVSIEMHHRQLSEAVAGSIVGVNVKGVGAKDLKRGFVISDPQKDPARGTKSFTAEMVVLQHPGQIRPGYVPVLHCHTSQVACNLVAITQKIDRETGKVFEENPEFVVAGDACTVEFRPTNPMAVEVFQQYPTLGRFMVRDLNQIVAVGIVKSVKRVM

>Spizellomyces punctatus EF-1alpha

MKTHVNLVVVGHLGSGKSTVLGHLMYKCGAIDKKDFDKIEWEAMKKDKPELKYAWILDKLREERERGTTIDIACDKLETPHRFVTVSDAPGHKDFIKNLITGTSQADCALLVVSAAVNEYETGIAREGQTREHALLAHTMGVRQLIVAVNKMDQVNWSQERFEKVQKELSDMMEKIGYKPESVPFVPVSGWTGDNLVDPSPNLPWFSRWKKQTKSGAVEGKTLLDAIDTIEPPIRPKDKPLRLPIQDVFKLSDIGTVAVGKVETGVLHPGKKVLLTPGGITTIVKSIQMHHEPLNEALPGDNVGLSVDLSIRDIHRGMVVSNPKDEPAKEAASFIAQIIILELPGRIEVGYTPVIDVHTSHVACKFDELIEKVDRRSGRTIAEHPKFLQMGDAAIVKLKPTKPLVVEAYADYPALGRFAMRDMRVTIAVGIVKSVEKVSGADIQSI
